# A phenome-wide approach to identify causal risk factors for deep vein thrombosis

**DOI:** 10.1186/s12920-023-01710-9

**Published:** 2023-11-11

**Authors:** Andrei-Emil Constantinescu, Caroline J. Bull, Lucy J. Goudswaard, Jie Zheng, Benjamin Elsworth, Nicholas J. Timpson, Samantha F. Moore, Ingeborg Hers, Emma E. Vincent

**Affiliations:** 1 MRC Integrative Epidemiology Unit at the University of Bristol, Oakfield House, Oakfield Grove, Bristol, UK; 2 Bristol Medical School, Population Health Sciences, University of Bristol, Oakfield House, Oakfield Grove, Bristol, UK; 3 School of Translational Health Sciences, Bristol Medical School, University of Bristol, Oakfield House, Oakfield Grove, Bristol, UK; 4 Health Data Research UK. Registered Office, 215 Euston Road, London, NW1 2BE UK; 5 School of Physiology, Pharmacology and Neuroscience, University of Bristol, Bristol, UK; 6 Department of Endocrine and Metabolic Diseases, Shanghai Institute of Endocrine and Metabolic Diseases, Ruijin Hospital, Shanghai Jiao Tong University School of Medicine, Shanghai, China; 7 Shanghai National Clinical Research Center for Metabolic Diseases, Key Laboratory for Endocrine and Metabolic Diseases of the National Health Commission of the PR China, Shanghai National Center for Translational Medicine, Ruijin Hospital, Shanghai Jiao Tong University School of Medicine, Shanghai, China; 8 Our Future Health Ltd. Registered office: 2 New Bailey, 6 Stanley Street, Manchester, M3 5GS UK; 9 UKRI Medical Research Council, Swindon, UK

**Keywords:** Mendelian randomization, Deep vein thrombosis, ALSPAC, Protein quantitative trait loci, Genome-wide association study

## Abstract

Deep vein thrombosis (DVT) is the formation of a blood clot in a deep vein. DVT can lead to a venous thromboembolism (VTE), the combined term for DVT and pulmonary embolism, a leading cause of death and disability worldwide. Despite the prevalence and associated morbidity of DVT, the underlying causes are not well understood. Our aim was to leverage publicly available genetic summary association statistics to identify causal risk factors for DVT. We conducted a Mendelian randomization phenome-wide association study (MR-PheWAS) using genetic summary association statistics for 973 exposures and DVT (6,767 cases and 330,392 controls in UK Biobank). There was evidence for a causal effect of 57 exposures on DVT risk, including previously reported risk factors (e.g. body mass index—BMI and height) and novel risk factors (e.g. hyperthyroidism and varicose veins). As the majority of identified risk factors were adiposity-related, we explored the molecular link with DVT by undertaking a two-sample MR mediation analysis of BMI-associated circulating proteins on DVT risk. Our results indicate that circulating neurogenic locus notch homolog protein 1 (NOTCH1), inhibin beta C chain (INHBC) and plasminogen activator inhibitor 1 (PAI-1) influence DVT risk, with PAI-1 mediating the BMI-DVT relationship. Using a phenome-wide approach, we provide putative causal evidence that hyperthyroidism, varicose veins and BMI enhance the risk of DVT. Furthermore, the circulating protein PAI-1 has a causal role in DVT aetiology and is involved in mediating the BMI-DVT relationship.

**Supplementary Information:**

The online version contains supplementary material available at 10.1186/s12920-023-01710-9.

## Introduction

Under normal physiological conditions, platelets and fibrin form clots to prevent blood loss at the site of vessel injury [1]. However, when clots (or thromboses) form abnormally they can disrupt blood flow [2, 3] and when this occurs in the deep veins of the limbs or pelvis this is known as deep vein thrombosis (DVT). A complication of DVT is pulmonary embolism (PE), where a clot breaks away from a deep vein wall and becomes lodged in a pulmonary blood vessel, obstructing blood flow to the lungs and causing respiratory dysfunction. In 2021, there were approximately one million incident cases of venous thromboembolism (VTE) in the United states alone [4]. DVT accounts for approximately two-thirds of VTE events and PE is the primary contributor to mortality. While VTE was a primary cause for 10,511 deaths in the UK in 2020 [5], the actual contribution of VTE to annual deaths is estimated to be 2–threefold higher [6].

To prevent acute and chronic complications it is essential to establish an accurate diagnosis of DVT. The symptoms of DVT alone are often not specific or sufficient to make a diagnosis, and about half of those suffering DVT will have no symptoms [7]. Symptoms are considered in conjunction with known risk factors to help estimate the likelihood of DVT and determine whether thromboprophylaxis is required [3]. Pharmacological thromboprophylaxis includes the use of anticoagulants, such as intravenous heparin and oral warfarin (a vitamin K antagonist), which have been used in combination to treat DVT for over 50 years, but require constant maintenance and monitoring [3]. More recently direct oral anticoagulants (DOAC), such as ﻿dabigatran (which inhibits thrombin) or rivaroxaban (which inhibits factor Xa), have been employed with reduced economic costs relative to traditional treatments [8].

Risk factors for DVT include age, obesity and genetic factors (such as deficiencies in the anticoagulation proteins: antithrombin, protein C, protein S and Factor V Leiden) [2, 9, 10]. However, the mechanisms through which these risk factors act have not been clearly established. The identification of novel causal risk factors and potential drug targets is required for improved DVT prophylaxis [3].

Mendelian randomization (MR) allows us to infer causality while addressing limitations of observational epidemiology such as confounding and reverse causation [11–14]. The design of a MR analysis is analogous to that of a randomised control trial (RCT), the “gold standard” method for evaluating the effectiveness of an intervention (Supplementary Fig. 1) [15]. It is an instrumental variable-based method that uses genetic variants as proxies (or instruments) for exposures to permit causal inference when interpreting relationships between these exposures and disease outcomes [16]. Here, we have used two-sample MR, which uses data from separate genome-wide association studies (GWAS) for exposures and outcomes of interest [17] to consider the effect of multiple exposures (phenotypes) on DVT risk.

To advance our understanding of DVT aetiology, we undertook a MR phenome-wide association study (MR-PheWAS). As 24 out of 57 exposures estimated to influence DVT were adiposity-related, we explored whether levels of circulating proteins, known to be altered by adiposity, were responsible for this association.

## Methods

### Study design

**Fig. 1:**
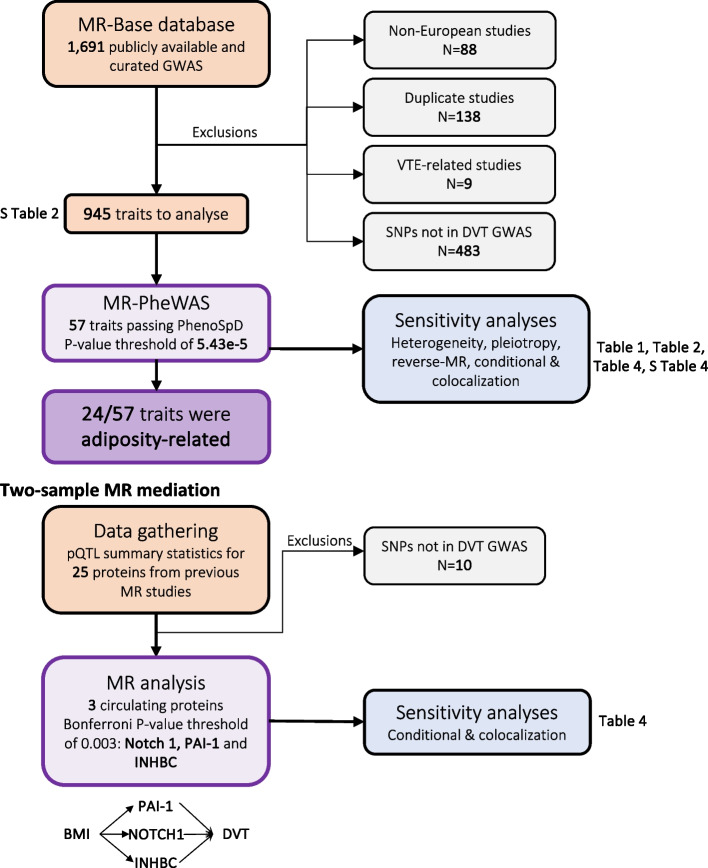
Overview of the study. First, a MR-PheWAS analysis to find risk factors for DVT was done using the MR-Base database and identified many of these to be associated with adiposity (*N*=24/57). This was followed by a two-sample mediation MR between BMI-associated pQTL data on DVT risk. MR = mendelian randomization; GWAS = genome-wide association study; VTE = venous thromboembolism; DVT = deep vein thrombosis; SNP = single-nucleotide polymorphism; pQTL = protein quantitative trait loci; PAI-1 = Plasminogen activator inhibitor-1; NOTCH1 = Neurogenic locus notch homolog protein 1; INHBC = Inhibin Subunit Beta C; S Table = Supplementary Table

With the aim to identify novel risk factors for DVT, we performed a MR-PheWAS to estimate the effects of 973 exposures on DVT risk. As 24 of the 57 exposures estimated to influence DVT were adiposity-related (see Table 1), we next decided to investigate potential mediators of this mechanistic relationship further. We focussed our mechanistic investigations on circulating proteins altered by adiposity [18, 19] and performed a two-sample mediation MR to estimate the effect of BMI on DVT with BMI-associated proteins as mediators. An overview of the study design is shown in Fig. 1. All analyses were conducted using R version 3.6.1. The MR-PheWAS was conducted using the TwoSampleMR R package [14]. STROBE-MR [20] reporting guidelines were followed (Additional file 4).

**Table 1:** Traits passing the PhenoSpD significance threshold (5.43E-5) in the MR-PheWAS of all traits in UK Biobank on DVT risk with the Inverse Variance Weighted (SNP > 1) and Wald Ratio (SN*P* = 1). Exposures highlighted in orange are referred to as "adiposity-related" in the main text

Exposure	No. SNP	MR method	Log Risk Ratio*	CI (95%)		SE	*P*-value	P_Het (ML)_	P_Plt_
**Treatment/medication code: warfarin**	7	IVW	4.29	3.09	5.49	0.61	1.40E-09	5.66E-40	0.4260
**Mania/bipolar disorder/manic depression**	1	WR	3.95	2.60	5.30	0.69	5.18E-06	NA	NA
**Chronic obstructive airways disease/copd**	1	WR	3.72	1.39	4.37	0.76	9.21E-07	NA	NA
**Treatment/medication code: carbimazole**	9	IVW	3.60	2.70	4.50	0.46	2.41E-12	5.21E-01	0.1048
**Varicose veins**	2	IVW	3.40	2.31	4.49	0.56	5.13E-07	4.42E-01	NA
**Hyperthyroidism/thyrotoxicosis**	6	IVW	2.39	1.88	2.90	0.26	8.69E-18	6.69E-01	0.3874
**Varicose veins of lower extremities**	16	IVW	1.90	1.30	2.50	0.31	2.36E-07	1.91E-01	0.5039
**Lysine**	1	WR	1.50	0.61	1.96	0.34	1.25E-05	NA	NA
**Prospective memory result**	2	IVW	1.46	1.02	1.90	0.23	5.33E-08	4.61E-01	NA
**Long-standing illness disability or infirmity**	14	IVW	1.25	0.87	1.63	0.20	8.13E-08	2.17E-01	0.4463
**Taking other prescription medications**	10	IVW	1.17	0.79	1.55	0.20	1.36E-06	4.83E-01	0.4399
**Eicosapentaenoate (EPA; 20:5n3)**	1	WR	1.10	0.75	1.45	0.18	3.14E-07	NA	NA
**Stearidonate (18:4n3)**	1	WR	1.09	0.73	1.45	0.18	1.22E-06	NA	NA
**Docosapentaenoate (n3 DPA; 22:5n3)**	1	WR	1.08	0.47	1.46	0.25	2.01E-05	NA	NA
**Adrenate (22:4n6)**	1	WR	1.01	0.55	1.32	0.20	3.48E-07	NA	NA
**X-14473**	1	WR	1.01	0.48	1.35	0.22	5.14E-06	NA	NA
**Qualifications: None of the above**	64	IVW	0.99	0.72	1.26	0.14	2.03E-10	6.18E-01	0.0335
**Arachidonate (20:4n6)**	1	WR	0.91	0.61	1.22	0.16	2.08E-06	NA	NA
**Overall health rating**	54	IVW	0.80	0.61	0.99	0.10	4.40E-14	5.14E-01	0.6398
**Leg fat percentage (right)**	246	IVW	0.59	0.47	0.71	0.06	3.32E-18	2.87E-03	0.2399
**Comparative body size at age 10**	157	IVW	0.57	0.46	0.68	0.06	3.98E-22	5.18E-01	0.1954
**Arm fat percentage (right)**	234	IVW	0.55	0.42	0.68	0.07	8.48E-14	8.47E-17	0.6940
**Arm fat percentage (left)**	253	IVW	0.55	0.41	0.68	0.07	1.61E-12	1.32E-24	0.6983
**Leg fat percentage (left)**	248	IVW	0.54	0.40	0.67	0.07	1.76E-12	7.00E-04	0.7261
**Leg fat mass (right)**	282	IVW	0.53	0.44	0.62	0.05	4.23E-28	9.07E-03	0.4978
**Leg predicted mass (right)**	361	IVW	0.52	0.43	0.60	0.04	8.79E-29	1.34E-02	0.6652
**Leg predicted mass (left)**	356	IVW	0.52	0.43	0.60	0.05	2.99E-27	5.18E-03	0.8052
**Body fat percentage**	253	IVW	0.51	0.41	0.61	0.05	1.48E-20	4.79E-02	0.6346
**Leg fat-free mass (left)**	361	IVW	0.51	0.42	0.60	0.05	6.10E-27	4.73E-03	0.8069
**Leg fat-free mass (right)**	363	IVW	0.50	0.41	0.59	0.05	1.11E-25	5.05E-03	0.5560
**Waist circumference**	227	IVW	0.50	0.40	0.59	0.05	1.74E-22	1.65E-02	0.5222
**Leg fat mass (left)**	281	IVW	0.50	0.40	0.59	0.05	1.85E-23	3.71E-02	0.5530
**Weight**	337	IVW	0.46	0.38	0.54	0.04	1.93E-28	1.33E-03	0.8573
**Arm fat mass (right)**	270	IVW	0.45	0.38	0.52	0.04	1.06E-30	3.60E-01	0.2818
**Arm fat mass (left)**	268	IVW	0.45	0.38	0.53	0.04	4.98E-29	1.93E-01	0.1348
**Basal metabolic rate**	377	IVW	0.45	0.36	0.54	0.05	2.62E-20	3.71E-03	0.7064
**Arm predicted mass (left)**	349	IVW	0.45	0.34	0.55	0.05	3.37E-14	1.53E-05	0.2577
**Trunk fat percentage**	237	IVW	0.44	0.35	0.54	0.05	2.91E-16	2.43E-03	0.6180
**Whole body fat mass**	280	IVW	0.44	0.36	0.51	0.04	4.65E-27	1.75E-01	0.1772
**Arm fat-free mass (right)**	350	IVW	0.44	0.33	0.54	0.05	1.66E-13	2.95E-04	0.2180
**Arm predicted mass (right)**	364	IVW	0.43	0.32	0.54	0.05	6.96E-13	9.35E-05	0.2660
**Trunk fat mass**	283	IVW	0.43	0.35	0.51	0.04	1.73E-23	2.90E-03	0.6360
**Arm fat-free mass (left)**	355	IVW	0.42	0.32	0.53	0.05	1.84E-12	3.14E-05	0.1920
**Whole body water mass**	405	IVW	0.42	0.32	0.51	0.05	7.67E-15	1.32E-04	0.3436
**Whole body fat-free mass**	405	IVW	0.41	0.31	0.50	0.05	3.90E-14	2.06E-04	0.3422
**Body mass index (BMI)**	305	IVW	0.40	0.32	0.47	0.04	1.60E-22	6.81E-02	0.5286
**Trunk fat-free mass**	406	IVW	0.39	0.29	0.48	0.05	2.32E-11	2.46E-06	0.0575
**Trunk predicted mass**	406	IVW	0.38	0.28	0.48	0.05	4.10E-11	9.09E-06	0.0513
**Hip circumference**	282	IVW	0.36	0.28	0.45	0.04	2.22E-13	2.92E-04	0.0876
**Comparative height size at age 10**	364	IVW	0.30	0.20	0.40	0.05	1.93E-06	1.56E-05	0.1080
**Overweight**	14	IVW	0.28	0.18	0.38	0.05	3.07E-05	3.44E-01	0.1711
**Obesity class 1**	17	IVW	0.18	0.11	0.25	0.03	1.34E-07	7.33E-01	0.2392
**Standing height**	591	IVW	0.17	0.09	0.24	0.04	4.61E-06	3.14E-05	0.1018
**Obesity class 2**	11	IVW	0.17	0.11	0.22	0.03	2.79E-06	5.45E-01	0.6859
**Height**	367	IVW	0.15	0.08	0.21	0.03	5.92E-06	1.58E-03	0.3372
**Impedance of leg (right)**	319	IVW	-0.55	-0.80	-0.35	0.12	2.21E-06	4.23E-06	0.0003
**Impedance of leg (left)**	323	IVW	-0.69	-1.05	-0.43	0.16	1.00E-05	9.96E-21	0.0072

^*^Methods: Inverse Variance Weighted (SNP > 1) and Wald Ratio (SN*P* = 1)

^*^LogRiskRatio is the logged value of the beta coefficient of the MR analysis into risk ratios. It can be read as an increase in the LogRisk of DVT per unit increase in trait

^*^PHET ML is the *P*-value of the Maximum Likelihood analysis looking at heterogeneity between genetic variants used to instrument a trait. H0 is that there is no heterogeneity present

^*^PPlt is the *P*-value of the MR-Egger analysis looking at the presence of horizontal pleiotropy. H0 is that there is no pleiotropy present

### Data preparation

#### Deep vein thrombosis GWAS data

Our outcome of interest (DVT) was presented in MR-Base as “Non-cancer illness code self-reported: deep venous thrombosis (dvt)”; these summary results describe a GWAS of Europeans (6,767 cases and 330,392 controls) performed using the PHEnome Scan ANalysis Tool (PHESANT), followed by genotypic data selected through SNP quality control (QC) [21, 22] (http://www.nealelab.is/uk-biobank).

#### GWAS data for exposures

Genetic data for exposures were obtained from the MR-Base platform of harmonised GWAS summary data [14]. The MR-Base platform permits the hypothesis-free analysis of all catalogued exposures to DVT. The exposures encompassed lifestyle, disease and biological traits. Non-European (*N* = 88) and duplicate (*N* = 138) studies were excluded. In the case of duplicate studies, those with the highest sample size were retained. VTE (DVT and PE) and VTE-related (e.g. phlebitis and thrombophlebitis) traits were removed (*N* = 9). The genetic instruments used for the analysis were single-nucleotide polymorphisms (SNPs) associated with each of the exposures at a genome-wide level of significance (*P* < 5e-8). As genetic confounding may bias MR estimates if SNPs are correlated [23], linkage disequilibrium (LD) clumping in PLINK [24] was conducted to ensure the SNPs used to instrument exposures were independent (radius = 10,000 kb; *r*^2^ = 0.001) using the 1000 Genomes European reference panel [25]. We also used the 1000 Genomes European dataset [25] to identify potential SNP proxies (with which the initial SNP is in LD with, *r*^2^ > 0.8) for those SNPs not present in the DVT summary statistics. Where not specified in Supplementary Table 2, the reported effect size for a given SNP was expressed along with the standard error (SE) in standard deviation units of the level of the risk factor for a continuous exposure, or as a unit change in the exposure on the log-odds scale for a binary trait.

#### Protein quantitative trait locus data

We aimed to determine whether BMI-associated proteins were mediating the relationship between adiposity and DVT. A list of BMI-associated proteins was obtained from two previous MR studies investigating the effect of BMI on the circulating proteome [18, 19]. We used protein quantitative trait loci (pQTL) data [26, 27] to identify SNPs associated with circulating protein levels at a genome wide level of significance (P ≤ 5e-08). Protein detection platforms for the pQTL data included the SOMAScan® by SomaLogic and Olink (ProSeek CVD array I) [28–31]. Twenty-five proteins were identified using these criteria (Supplementary Table 1). PLINK clumping (radius = 10,000 kb; *r*^2^ = 0.001) was performed to ensure the genetic variants used to instrument protein levels were independent. Proxy SNPs for those SNPs that were not present in the DVT data were identified through the 1000 Genomes European dataset [25].

#### Data harmonisation

The majority of GWAS present the effects of a SNP on a trait in relation to the allele on the forward strand. However, the allele present on the forward strand can change as reference panels get updated. This requires correction (harmonisation) so that both exposure and outcome data reference the same strand [32]. For exposure and outcome data harmonisation, incorrect but unambiguous alleles were corrected, while ambiguous alleles were removed. In the case of palindromic SNPs (A/T or C/G), allele frequencies were used to solve ambiguities. Harmonisation was not possible for 483 exposures (variants were not present in the DVT GWAS), resulting in a final list of 973 exposures to include in the MR-PheWAS (Supplementary Table 2). For our pQTL analysis, 21 out of 25 proteins had genetic variants (including proxies) available in the DVT GWAS, and only 15 proteins had valid SNPs after harmonization (Supplementary Table 3). Finally, PhenoSpD was used for multiple testing correction in the MR-PheWAS analysis (*P* = 5.43e-5), while Bonferroni correction was used in the pQTL MR (*P* = 0.003) (Supplementary Methods).

#### MR-PheWAS

A hypothesis-free MR-PheWAS was conducted using the TwoSampleMR R package [33]. The effect of a given exposure on DVT was estimated using the inverse-variance weighted (IVW) method for exposures with more than one SNP [34]. Wald ratios (WRs) were derived for exposures with a single SNP [35]. A full description of all MR analyses referenced in this study is available in the Supplementary Methods, while SNPs used in the MR analysis are available in Supplementary Table 5.

#### Conditional analysis

We performed a conditional analysis for each single-SNP trait using the GCTA-COJO software [36] to identify any potential shared secondary signals in a 1 MB region [37], with the aim of performing an additional colocalization analysis on those secondary signals if the primary colocalization analysis did not find a shared causal signal. We downloaded summary statistics for these traits from OpenGWAS (https://gwas.mrcieu.ac.uk/) [38] and used genotypic data from the Avon Longitudinal Study of Parents and Children (ALSPAC) as a reference panel. Further details of the cohort are described elsewhere [39, 40], in brief: 14,541 pregnancies to women with an expected delivery date of April 1, 1991, to December 31, 1992, were enrolled. We used the genotypic data of 8,890 mothers to perform our conditional analysis. Ethical approval for the study was obtained from the ALSPAC Ethics and Law Committee and the Local Research Ethics Committee. The study website contains details of all available data through a fully searchable data dictionary and variable search tool (http://www.bristol.ac.uk/alspac/researchers/our-data/).

#### Colocalization analysis

Only one genetic instrument was available for some of the exposures investigated (*N* = 10). As the Wald ratio estimator is susceptible to genetic confounding, we performed a colocalization analysis on the un-pruned genetic dataset for each single-SNP trait. Genetic confounding in this case refers to confounding by LD, where the SNP associated with the exposure is in LD with a SNP affecting another trait that affects the outcome independent of the exposure, which invalidates MR assumptions [41]. Colocalization analysis uses Bayesian statistics to estimate whether an exposure and outcome share a causal signal in a region of the genome [42], which can then strengthen the evidence that there is a causal relationship by providing evidence that the detected effect in the MR analysis is not due to confounding by LD. We used the R package “coloc” (https://cran.r-project.org/web/packages/coloc/) approximate Bayes factor (coloc.abf) function with default settings for prior probabilities to conduct a colocalization analysis with the following hypotheses: H0 (no causal variant), H1 (causal variant for trait 1 only), H2 (causal variant for trait 2 only), H3 (two distinct causal variants) and H4 (one common causal variant) [42]. We then used LocusZoom (https://locuszoom.org/) to provide visual evidence for the presence of a shared signal between our exposures and DVT.

## Results

### MR-PheWAS

**Fig. 2:**
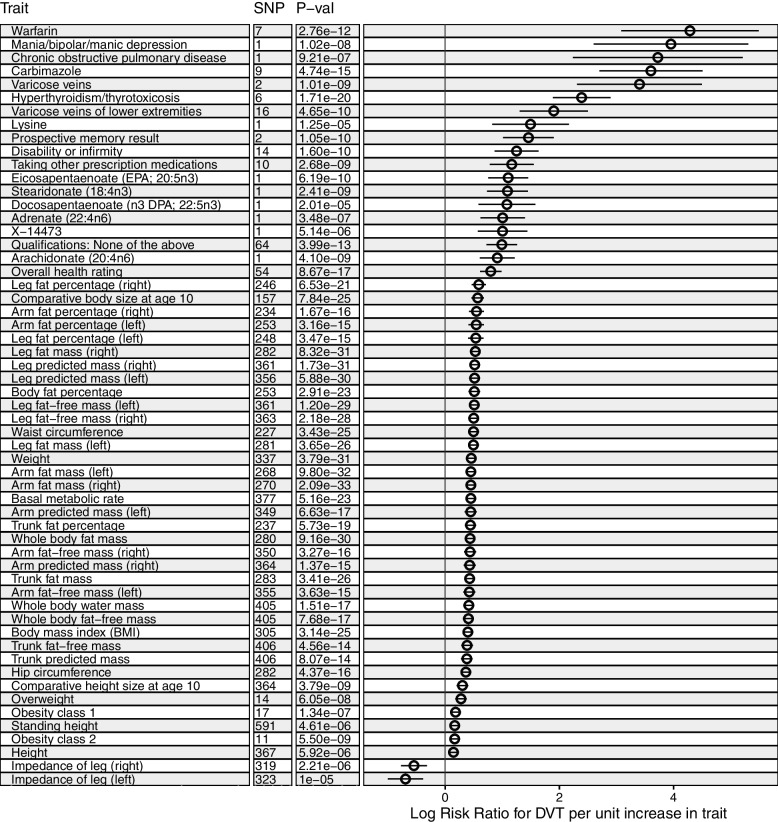
A many-to-one forest plot of the exposures which passed the *P*-value threshold following multiple testing correction (5.43e-5). Each trait is accompanied by two additional descriptive columns (No. SNPs and *P* -value), while log risk ratio (RR) is displayed to the right, alongside with the confidence intervals. MR methods: Inverse variance weighted (SNP > 1) and Wald ratio (SNP = 1)

Of the 973 exposures investigated, 945 were identified as independent using PhenoSpD, setting the *P*-value threshold for our MR analysis at 5.43e-5. Fifty-seven exposures were estimated to influence DVT risk (Fig. 2, Table 1). Sensitivity analyses results for all traits using additional MR methods are shown in Supplementary Table 4.

We observed strong causal evidence for a number of exposures including: “Hyperthyroidism/thyrotoxicosis” (IVW Log RR: 2.39, 95% CI: 1.88 to 2.90; *P* = 8.69e-18); “Treatment/medication code: carbimazole” (IVW Log RR: 3.60, 95% CI: 2.70 to 4.50, *P* = 2.41e-12); “Chronic obstructive airways disease/chronic obstructive pulmonary disease (COPD)” (WR Log RR: 3.72, 95% CI: 1.39 to 4.37; *P* = 9.21e-07); “Varicose veins” (IVW Log RR: 1.90, 95% CI: 1.30 to 2.50; *P* = 2.36e-07) and “Varicose veins of the lower extremities” (IVW Log RR: 3.40, 95% CI: 2.31 to 4.49; *P* = 5.13e-07) (Fig. 2, Table 1).

Adiposity, an established risk factor for DVT [43], and its related traits (*N* = 24, see Table 1 note) were all positively associated with DVT. These include traits identified in previous MR studies, such as “Body Mass Index” (IVW Log RR: 0.40, 95% CI: 0.32 to 0.47; *P* = 1.60e-22), fat mass e.g. “Whole body fat mass” (IVW Log RR: 0.44, 95% CI: 0.36 to 0.51; *P* = 4.65e-27) and fat-free mass e.g. “Whole body fat-free mass” (IVW Log RR: 0.41, 95% CI: 0.31 to 0.50; *P* = 3.90e-14) [44] (Fig. 2, Table 1). Another previously-associated trait is “Height” (IVW Log RR: 0.15, 95% CI: 0.08 to 0.21; *P* = 5.92e-06) [45]. Other associated height-related traits not previously investigated in an MR framework include “Standing height” (IVW Log RR: 0.17, 95% CI: 0.09 to 0.24; *P* = 4.61e-06) and “Comparative height size at age 10” (IVW Log RR: 0.30, 95% CI: 0.20 to 0.40; *P* = 1.93e-06) (Fig. 2, Table 1).

Over 50% of the exposures (*N* = 31) which passed our *P*-value threshold for multiple testing were found to have heterogenous effects between instruments using the maximum likelihood method. Of these, most (*N* = 24) were traits related to body size (mass and adiposity). The remaining heterogenous traits were: “basal metabolic rate” (PHet: 3.71e-03); “warfarin treatment” (PHet: 5.66e-40); “Height” (PHet: 1.58e-03); “Standing height” (PHet = 4.61e-06); “Comparative height size at age 10” (PHet = 1.93e-06); “Impedance of leg (right)” (PHet: 4.23e-06) and “Impedance of leg (left)” (PHet: 9.96e-21). These findings are consistent with our IVW and MR-Egger heterogeneity analyses (Table 1).

MR-Egger estimates indicated strong evidence of horizontal pleiotropy for “Qualifications: None of the above” (intercept = -5.69e-04, *P* = 3.35e-02), “Impedance of leg (right)” (intercept = 2.58e-04, *P* = 3.22e-04) and “Impedance of leg (left)” (intercept = 2.22e-04, *P* = 7.24e-03) (Table 1). The former trait refers to those who answered “None of the above” in the self-report questionnaire on education in UK Biobank (“College or University degree”, “A levels/AS levels or equivalent”, “O levels/GCSEs or equivalent”, “CSEs or equivalent”, “NVQ or HND or HNC or equivalent”, “Other professional qualifications eg: nursing, teaching”). We were unable to assess whether the “Prospective memory result” trait was pleiotropic, as this exposure was instrumented using only 2 SNPs. In bidirectional MR analyses, DVT was estimated to increase warfarin treatment (“Treatment/medication code: warfarin” (beta = 0.29; SE = 0.02; *P* = 1.79e-30)), implying reverse causation, and therefore violating MR assumptions (Table 2).

**Table 2:** Reverse MR of traits passing the *P*-value threshold from the main analysis in Table 1. Exposures highlighted in orange are referred to as "adiposity-related" in the main text

Outcome	No. SNP	MR method*	Beta*	SE	*P*-value	P_Het (ML)_	P_Plt_
**Treatment/medication code: warfarin**	9	IVW	0.29	0.02	3.81E-32	9.63E-02	5.11E-01
**Stearidonate (18:4n3)**	5	IVW	1.35	0.50	6.78E-03	9.11E-01	8.41E-01
**Leg predicted mass (left)**	9	IVW	0.51	0.23	2.73E-02	4.19E-04	6.13E-01
**Leg fat-free mass (left)**	9	IVW	0.50	0.23	2.86E-02	5.20E-04	6.10E-01
**Leg predicted mass (right)**	9	IVW	0.47	0.23	4.12E-02	3.95E-04	6.03E-01
**Long-standing illness disability or infirmity**	9	IVW	0.19	0.10	4.69E-02	2.25E-01	9.50E-01
**Leg fat-free mass (right)**	9	IVW	0.47	0.23	4.71E-02	3.47E-04	6.09E-01
**Taking other prescription medications**	9	IVW	0.16	0.10	8.84E-02	7.97E-01	2.34E-01
**Varicose veins**	9	IVW	0.02	0.01	9.89E-02	7.21E-01	3.19E-01
**Eicosapentaenoate (EPA; 20:5n3)**	5	IVW	0.58	0.44	1.87E-01	5.75E-01	8.16E-01
**Leg fat percentage (left)**	9	IVW	-0.35	0.27	1.91E-01	8.77E-07	9.80E-01
**Qualifications: None of the above**	9	IVW	-0.11	0.09	2.06E-01	1.02E-01	8.73E-01
**Varicose veins of lower extremities**	9	IVW	0.04	0.03	2.38E-01	2.08E-01	5.93E-01
**Weight**	9	IVW	0.22	0.25	3.62E-01	1.95E-02	5.78E-01
**Leg fat percentage (right)**	9	IVW	-0.25	0.28	3.80E-01	3.18E-07	9.90E-01
**Hyperthyroidism/thyrotoxicosis**	9	IVW	-0.01	0.02	3.89E-01	9.48E-01	6.27E-01
**Arm fat percentage (left)**	9	IVW	0.35	0.42	3.96E-01	6.65E-12	8.82E-01
**Arm fat percentage (right)**	9	IVW	0.34	0.41	4.15E-01	1.70E-11	8.55E-01
**Arm fat mass (left)**	9	IVW	0.31	0.39	4.21E-01	1.84E-05	7.77E-01
**Arachidonate (20:4n6)**	5	IVW	0.24	0.30	4.28E-01	8.53E-01	8.94E-01
**Hip circumference**	9	IVW	0.22	0.29	4.37E-01	8.58E-03	9.32E-01
**Basal metabolic rate**	9	IVW	0.21	0.28	4.54E-01	1.46E-06	6.68E-01
**Whole body water mass**	9	IVW	0.20	0.32	5.31E-01	1.38E-09	7.29E-01
**Whole body fat-free mass**	9	IVW	0.20	0.32	5.42E-01	1.22E-09	7.16E-01
**Waist circumference**	9	IVW	0.12	0.25	6.24E-01	1.70E-02	9.81E-01
**Obesity class 2**	5	IVW	1.20	2.53	6.33E-01	7.08E-01	5.15E-01
**Arm predicted mass (right)**	9	IVW	-0.13	0.32	6.91E-01	1.30E-10	8.85E-01
**Overweight**	5	IVW	-0.46	1.17	6.95E-01	6.26E-01	8.70E-01
**Trunk fat percentage**	9	IVW	0.18	0.45	6.98E-01	3.16E-09	9.39E-01
**Whole body fat mass**	9	IVW	0.13	0.36	7.18E-01	1.43E-04	6.77E-01
**Arm fat-free mass (right)**	9	IVW	-0.11	0.33	7.30E-01	9.99E-11	7.45E-01
**Arm predicted mass (left)**	9	IVW	-0.11	0.33	7.47E-01	1.21E-10	8.46E-01
**Comparative height size at age 10**	9	IVW	0.07	0.25	7.70E-01	2.37E-04	6.67E-01
**Treatment/medication code: carbimazole**	9	IVW	0.00	0.01	7.83E-01	3.46E-01	9.43E-01
**Arm fat-free mass (left)**	9	IVW	-0.09	0.32	7.92E-01	5.50E-10	8.24E-01
**Mania/bipolar/manic depression**	9	IVW	0.00	0.01	8.09E-01	4.14E-01	8.69E-01
**Trunk predicted mass**	9	IVW	0.07	0.39	8.67E-01	1.33E-15	7.36E-01
**Leg fat mass (right)**	9	IVW	-0.04	0.29	8.84E-01	1.75E-04	8.88E-01
**Trunk fat-free mass**	9	IVW	0.05	0.39	9.03E-01	1.59E-15	7.25E-01
**Body fat percentage**	9	IVW	0.04	0.37	9.06E-01	5.73E-09	8.91E-01

^*^Method: Inverse variance weighted (IVW)

^*^Beta column represents the effect estimate from the MR analysis of DVT on trait risk

### Estimated effects of BMI-driven proteins on DVT risk

**Fig. 3:**
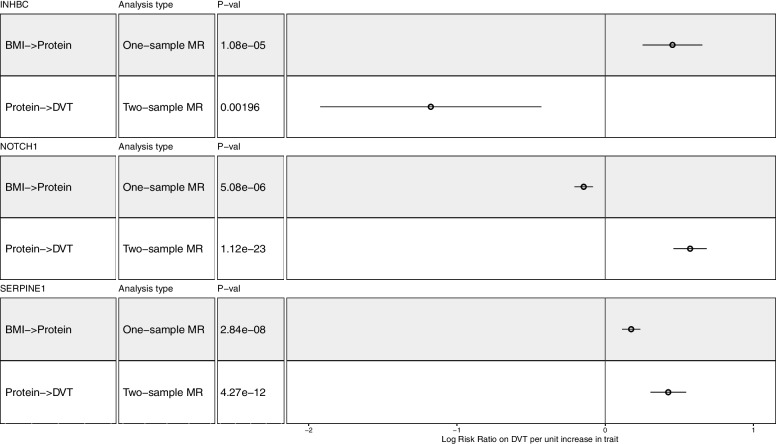
A many-to-one forest plot of the three BMI-associated proteins which passed the multiple-testing corrected *P*-value threshold (0.003) in the MR analysis. Each protein is accompanied by two additional descriptive columns (type of analysis conducted and *P*-value), while the effect is displayed to the right, alongside with the confidence intervals (Beta coefficient/Log RR ± 95% CI). Effect sizes of BMI on proteins taken from Goudswaard et al. [18] and Zaghlool et al. [19]

Of the 57 traits estimated to increase risk of DVT (Table 1, Fig. 2), 24 were adiposity-related. While adiposity is an established risk factor for DVT, the biological mechanisms underlying the effect of adiposity on DVT are not well understood. We therefore used a two-sample MR mediation analysis to test whether altered levels of 15 circulating blood proteins, driven by adiposity, are responsible for this association. Two recent MR studies have demonstrated that BMI causally affects the levels of 15 circulating proteins [18, 19]. Three of these proteins were estimated to influence DVT risk: Neurogenic locus notch homolog protein 1 (NOTCH1; WR Log RR: 0.57, 95% CI: 0.45 to 0.68; *P* = 1.12e-23), Plasminogen activator inhibitor-1 (PAI-1; WR Log RR: 0.42, 95% CI: 0.30 to 0.54; *P* = 4.27e-12) and Inhibin beta C chain (INHBC; WR Log RR: -1.18, 95% CI: -2.18 to -0.69; *P* = 0.002). Mediation analysis was performed for PAI-1 (the only protein where BMI-protein and protein-DVT effect estimates were consistent in directionality): the proportion of the BMI-DVT effect mediated by PAI-1 was estimated to be 18.56% (Table 3, Fig. 3, Supplementary Table 3).

**Table 3:** Mediation MR analysis of BMI-associated protein levels on DVT passing the multiple testing *P*-value threshold (0.003), with a two-step MR of the indirect effect of BMI on DVT through protein levels and proportion mediated (%) by PAI-1

Exposure	MR method	Log Risk Ratio*	CI (95%)		*P*-value	Beta coefficient—BMI to protein*	Proportion (%) mediated by protein
Neurogenic locus notch homolog protein 1	Wald ratio	0.57	0.45	0.68	1.12E-23	-0.15	Effect not consistent
Plasminogen activator inhibitor 1	Wald ratio	0.42	0.30	0.54	4.27E-12	0.17	18.56
Inhibin beta C chain	Wald ratio	-1.18	-2.18	-0.69	1.96E-03	0.45	Effect not consistent

^*^LogRiskRatio is the logged value of the beta coefficient of the MR analysis into risk ratios. It can be read as an increase in the LogRisk of DVT per increase in cirulating protein levels

^*^BMI-Protein MR effect estimates from Goudswaard et al. (https://doi.org/10.1038/s41366-021-00896-1) and Zaghlool et al. (https://doi.org/10.1038/s41467-021-21542-4)

### Conditional and colocalization analyses

**Fig. 4:**
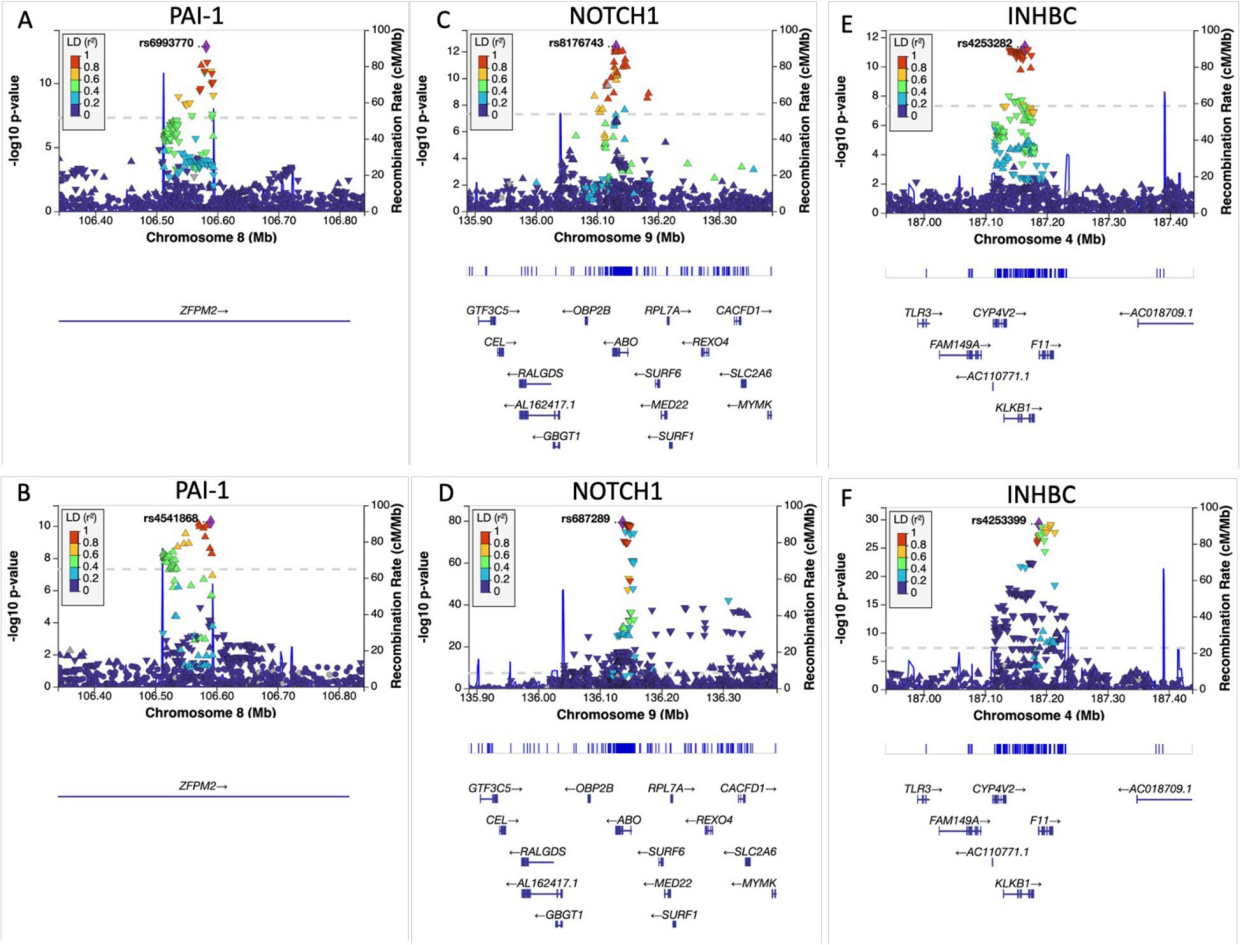
LocusZoom plots in a 1Mb region of the SNP used to proxy each PAI-1 in both exposure (**A**) and outcome (DVT, **B**) data. The x-axis represents the position within the chromosome, while the y-axis is the -log_10_ of the *P*-value. Each dot is a SNP, and the colours indicate how much LD there is between the reference SNP and the other genetic variants

Seven of the 57 traits in the MR-PheWAS and 3 proteins from the pQTL MR analyses could be instrumented using only one genetic variant, and therefore required a conditional and colocalization analysis to provide additional evidence of causality. There were no secondary signals after conditioning on the top SNP for each exposure-DVT pair. There was evidence of a shared causal variant for PAI-1 (PP.S = 97.5%), strengthening the evidence that there is a true causal relationship between the levels of this protein and DVT (Table 4, Fig. 4). For the other traits, this indicated that we couldn’t be certain that the effect seen in the MR is not due to confounding by LD, which as opposed to the PAI-1 findings, limits the evidence of a causal effect of those traits on DVT.

**Table 4:** Colocalization analysis results for exposures instrumented through only one SNP

Analysis type	Exposure	nr SNP*	PP.H0	PP.H1	PP.H2	PP.H3	PP.H4
BMI-associated proteins	**Plasminogen activator inhibitor 1**	2604	3.0254E-13	1.9614E-06	3.9637E-09	0.02472248	0.97527556
	**Neurogenic locus notch homolog protein 1**	3856	1.0694E-79	4.778E-73	2.2382E-07	0.99999972	6.0801E-08
	**Inhibin beta C chain**	4079	1.1109E-29	2.6137E-23	4.2502E-07	0.99999948	9.3591E-08
MR-PheWAS	**Lysine**	547	2.4588E-11	0.98338278	3.2772E-13	0.01310352	0.0035137
	**Bipolar disorder / mania**	3533	0.47264348	0.43702738	0.03965284	0.03665077	0.01402554
	**Chronic obstructive pulmonary disorder**	4229	0.0766326	0.83975957	0.00333097	0.03645779	0.04381907
	**X-14473**	655	6.292E-07	0.83967623	6.959E-08	0.09280245	0.06752062
	**Docosapentaenoate**	614	1.9077E-08	0.62830917	1.1181E-09	0.03649044	0.33520037
	**Adrenate**	626	1.8886E-18	0.5838098	1.1167E-19	0.03413747	0.38205274
	**Stearidonate**	674	5.34E-11	0.50441818	3.2335E-12	0.03007888	0.46550294
	**Eicosapentanoate**	633	2.8064E-17	0.22721212	1.6606E-18	0.01268473	0.76010315
	**Arachidonate**	626	4.9721E-77	0.17796851	2.9399E-78	0.00971061	0.81232088

Posterior probabilities for: H0 (no causal variant), H1 (causal variant for trait 1 only), H2 (causal variant for trait 2 only), H3 (two distinct causal variants) and H4 (one common causal variant)

^*^nr SNPs are the number of SNPs in the 500 kb genomic window used to run the colocalization analysis

## Discussion

With the aim to identify novel causal risk factors for DVT, we performed a hypothesis-free MR-PheWAS of 945 exposures to DVT, of which 57 passed a conservative *P*-value threshold for evidence of causality. We confirmed causality for several previously established risk factors for DVT (such as BMI and height) and have identified several novel putative causal risk factors (such as hyperthyroidism and varicose veins). Of the 57 exposures estimated to influence DVT risk, 24 were adiposity-related traits. Therefore, we investigated whether the impact of adiposity on DVT is mediated by circulating proteins known to be altered by BMI [18, 19]. Here, we provide novel evidence that the circulating protein, PAI-1 has a causal role in DVT aetiology and is involved in mediating the BMI-DVT relationship.

Height has been previously associated with increased DVT risk [46] and our results align with this finding. With increased height, a greater volume of blood is required which can increase the stress on blood vessels, disrupting haemostasis [46]. Fat-free mass was also estimated to increase risk of DVT in our study. While counterintuitive, this effect could be mediated through height, as taller people usually have more fat-free mass [44, 45]. As expected, many body size related traits showed evidence of heterogeneity, likely due to the large number of SNPs used to instrument these traits and the many underlying biological pathways explaining variation in adiposity.

Venous blood stasis caused by immobility is also a known risk factor for DVT [3]. Here, we report evidence that long standing illness, disability, or infirmity increases DVT risk. A proposed mechanism is stasis of blood flow in the veins which can be either due to a particular neurological condition or due to the paralysis of the lower limbs [47].

Our study also provides evidence for novel DVT risk factors. Hyperthyroidism has previously been proposed to contribute to DVT, as indicated by a recent systematic review and meta-analysis of cohort studies showing association with DVT (RR: 1.33, 95% CI: 1.28 to 1.39; I^2^ = 14%) [48]. In the present study, we provide novel evidence for a causal effect of hyperthyroidism/thyrotoxicosis on DVT risk (IVW RR: 10.91, 95% CI: 3.97 to 18.17; *P* = 3.14e-25). The underlying mechanism is not fully understood but may involve thyroid hormones (THs) promoting a hypercoagulable state and venous thrombi formation, by increasing plasma concentration of factor VIII, fibrinogen, PAI-1 and vWF [49]. TH T4 may also directly enhance platelet function through integrin α_v_β_3_ [50]. In addition, THs enhance basal metabolic rate (BMR) and thermogenesis, both of which affect body weight. Indeed, we found that an increase in basal metabolic rate is associated with DVT. While a higher BMR should lead to lower BMI and thus lower DVT risk, it is likely that our results may be explained by the hyperthyroidism-associated mechanisms outlined above.

Our MR estimates also support evidence of a causal association between varicose veins and increased risk of DVT. Varicose veins can result in the inability of the blood to fully return to the heart, leading to the enlargement of the veins, and in time, potentially an increased risk of DVT due to stasis [51]. Varicose veins have been outlined as a possible risk factor in general practice patients in Germany [52], as well as in a Chinese retrospective study of over 100 K people [51].

COPD was also associated with an increased risk of DVT. COPD is a severe chronic respiratory disease, having been studied extensively for its role in PE [53]. Indeed, both PE and DVT are more prevalent and underdiagnosed in people with COPD [54]. Our colocalization analysis did not provide evidence that would support our MR estimates. Moreover, as the SNP used to proxy for COPD (rs9579496) is intergenic i.e. in-between genes, we were unable to compare our results with any locus-specific experimental studies.

Finally, as adiposity is an established risk factor for DVT, the estimates we observe between adiposity-related traits and DVT most likely reflect true causal relationships. The estimate we report here for BMI (RR: 1.49, 95% CI: 1.38 to 1.60; *P* = 3.14e-25) is consistent with a previous MR study conducted in individuals of Danish descent (OR: 1.57, 95% CI: 1.08 to 1.97; *P* = 3e-03) [10]. In addition, our results are in agreement with the estimated effect of BMI on VTE in the FinnGen consortium (MR RR: 1.58, 95% CI: 1.28 to 1.95; *P* = 2.00e-05) [44]. Higher adiposity is associated with dysregulated metabolism, which is one factor that can promote a hypercoagulable state and impair venous return, increasing the chance of thrombi formation [55]. Given that 42% of the traits we found to be associated with DVT were adiposity-related, and that previously we and others found that adiposity is associated with changes to the circulating proteome [18, 19], we hypothesised that adiposity-driven changes to the circulating proteome may promote DVT. BMI-driven candidates include proteins that can modulate coagulation (anti-thrombin III, PAI-1) [56, 57], platelet function (adiponectin, IGFBP/IGF) [58] and/or thrombosis (galectin-3) [59].

Using our MR approach, we were able to estimate the effect of 15 BMI-driven circulating proteins on DVT risk. Our analyses suggest a causal role for 3 of these proteins (NOTCH1, PAI-1 and INHBC). Given the established role of some of the circulating proteins in coagulation and thrombosis, the lack of evidence for an estimated effect is surprising e.g. anti-thrombin III [56]. This could represent a true result or our ability to instrument circulating proteins using single SNPs.

PAI-1 was the only protein for which evidence was directionally consistent with mediation of the BMI-DVT relationship (circulating levels of PAI-1 were positively associated with BMI and with DVT). A study using data from the Million Veterans Program to identify novel VTE risk factors has also confirmed colocalization with DVT for the same PAI-1 SNP (rs6993770, *ZFPM2* locus) used in our analysis [60]. Klarin et al. previously identified in their MR analysis that rs4602861 (*ZFPM2* locus) increased the risk of VTE (OR: 1.08, CI: 1.03–1.15) [61], which is in LD with our PAI-1 SNP used here (*R*^2^ = 0.93). In addition to replicating this previous finding, we have also shown that this locus increases DVT risk through regulating PAI-1 levels. Moreover, PAI-1 has been associated with an increase in VEGF levels [62–64], which was found to increase the risk of VTE in a previous MR study [65], further adding to the evidence that PAI-1 is involved in DVT development. A follow-up analysis in a murine model found that PAI-1-overexpressing mice had 1.5-fold larger thrombus size compared to PAI-1^−/−^ mice [60]. Moreover, a recent observational study done in inhabitants of Tromsø, Norway (cases = 383, controls = 782) found that PAI-1 increased the risk of future VTE, and that PAI-1 mediated ~ 15% of the obesity-VTE relationship [66], a number comparable to our MR estimate (18.6%). These results are consistent with the known role for PAI-1 in inhibiting fibrinolysis (breakdown of a clot) [67]. In addition, PAI-1 expression has been previously found to be associated with DVT formation in mice [67] and in humans after total hip arthroplasty [57]. PAI-1 overexpression is enhanced in visceral fat tissue [68], and while waist-to-hip ratio (WHR) is highly correlated with visceral fat [69], we did not find evidence of an effect of WHR on DVT (Supplementary Table 4). Finally, there has been extensive research into PAI-1 drug targets, ranging from synthetic peptides, RNA aptamers to monoclonal antibodies [70]. Rosuvastatin, an HMG-CoA reductase inhibitor, has been found to inhibit PAI-1 in vitro [71]. Randomised clinical trials using rosuvastatin have confirmed that it reduced occurrence of symptomatic venous thromboembolism [72] and increased plasma fibrinolytic potential [73], supporting a role for statins in VTE treatment and prevention, possibly via altered PAI-1.

Although we found evidence for a role of INHBC and NOTCH1 in DVT risk, estimates were inconsistent with mediation of the BMI-DVT relationship. We found that circulating INHBC levels were negatively associated with DVT, suggesting circulating levels of INHBC may have a protective effect. Inhibins are part of the growth and differentiation superfamily of transforming growth factor beta (TGF-β) [74] and play a role in inhibiting the levels of follicle-stimulating hormone (FSH) produced by the pituitary gland [75]. Although we did not find evidence of causality between FSH and DVT, a recent study showed that FSH can enhance thrombin generation [76]. This discrepancy could be due to INHBC acting through a different pathway compared to FSH. With regards to NOTCH1, we found that higher expression was associated with an increased risk of DVT. NOTCH1 plays a role in responses to microenvironmental conditions, vascular development and is a shear stress and flow sensor in the vasculature [77]. While NOTCH targeting has not been done in relation to VTE, current small molecular drugs such as Crenigacestat [78] and targeting antibodies such as Brontictuzumab [79] are being used in clinical trials to inhibit NOTCH signalling for the treatment of T-cell acute lymphoblastic leukaemia and solid tumours, respectively [80]. Nevertheless, the pQTLs for these two proteins had a stronger association with DVT, and this might indicate reverse causation, horizontal pleiotropy or measurement error in the exposure (i.e. protein levels) [81, 82]. Therefore, the results for INHBC and NOTCH1 should be interpreted with caution, as the colocalization analysis did not provide evidence for a shared signal for the SNPs instrumenting these two proteins and DVT, which does make it more likely that these results are due to confounding by LD [41].

There are some limitations to our approach. Firstly, although the number of traits in MR-Base is large and continues to grow, and the approach was undertaken in a hypothesis-free manner, we were limited by the traits available in the platform at the time of the analysis. In addition, the availability of genetic instruments for some traits within the platform are limited, meaning a false null finding could be reported. While the number of exposures in OpenGWAS/MR-Base allows for a large analysis of aggregated data, this can also come at the cost of being limited by the GWAS data present in the database. For example, the COPD trait used here had only one instrument, while a more recent GWAS of COPD done in UKBB had identified 82 associations with COPD [83]. Moreover, some of the exposures did not have a SNP or proxy present in the outcome (DVT) dataset, making it infeasible to perform MR analysis. Finally, we have chosen to investigate risk factors for DVT as opposed to PE (which is observed in about 40% of DVT cases [84] to increase our power to detect causal risk factors. Future analyses could focus on PE specifically to identify predictive risk factors for this outcome.

In summary, we have confirmed estimates of previously identified traits on DVT (e.g. adiposity-related, height), and identified novel estimates (e.g. hyperthyroidism and varicose veins) with the disease. We also provide evidence that the relationship between adiposity and DVT is mediated by dysregulated levels of circulating proteins (PAI-1). These findings improve the understanding of DVT aetiology and have notable clinical significance, particularly in regard to hyperthyroidism and PAI-1.

### Supplementary Information

**Additional file 1: Supplementary Figure 1.** Mendelian randomization (MR) assumptions. MR works in a similar way to a randomized controlled trial, exploiting the essentially random allocation of alleles at conception and the independent assortment of parental variants at meiosis. MR uses genetic variants (G) as proxies (instruments) to investigate whether an exposure (E), is causally associated with a disease outcome (O), in this case DVT. E is causally associated with O if the following conditions are held: (1) the genetic variant (G) is a valid instrument, in that it is reliably associated with E; (2) there is no independent association with O, except through E; and (3) the instrument is independent of any measured or unmeasured confounding factors (C). **Supplementary Figure 2.** Many-to-one Forest plot of the BMI-associated proteins which passed the *P*-value threshold after multiple testing correction. Each protein is accompanied by four additional descriptive columns (GWAS author, MR method, No. SNPs and *P*-value), while log risk ratio (RR) is displayed to the right, alongside with the confidence intervals. MR methods: Inverse variance weighted (SNP > 1) and Wald ratio (SNP = 1). **Supplementary Figures 3-6.** LocusZoom plot of the 1MB region within the top SNP for the proteins which did not colocalize or pass the multiple testing adjusted *P*-value threshold. The top signal is displayed on the left for the pQTL data and on the right for the DVT data. The x-axis represents the position inside the chromosome, while the y-axis is the -log10 of the *P*-value. Each dot is a SNP, and the colours indicate the amount of LD between the reference SNP (top signal in the region) and the other genetic variants.

**Additional file 2: Supplementary Table 1.** Traits considered as exposures in the analysis of BMI-associated proteins on DVT. **Supplementary Table 2.** Traits considered as exposures in the hypothesis-free MR analysis. **Supplementary Table 3.** MR analysis of BMI-associated protein levels on DVT. **Supplementary Table 4.** Secondary hypothesis-free analysis of traits on DVT with additional MR methods (where possible). **Supplementary Table 5.** SNPs for traits used in the MR analyses.

**Additional file 3. **Supplementary Methods.

**Additional file 4. **STROBE-MR checklist of recommended items to address in reports of Mendelian randomization studies^1^ ^2^.

## Data Availability

Summary-level GWAS data used in this study are publicly available without the need for application through the MR-Base platform, which is accessible at http://www.mrbase.org/. Scripts used to perform the analyses in this study are available on GitHub at https://github.com/andrewcon/dvt-mr.

## References

[1] Baaten CCFMJ, Ten Cate H, Van Der Meijden PEJ, Heemskerk JWM (2017). Platelet populations and priming in hematological diseases. Blood Rev.

[2] Mackman N (2012). New insights into the mechanisms of venous thrombosis. J Clin Invest.

[3] Stone J, Hangge P, Albadawi H, Wallace A, Shamoun F, Knuttien MG (2017). Deep vein thrombosis: pathogenesis, diagnosis, and medical management. Cardiovasc Diagn Ther.

[4] Heart Disease and Stroke Statistics-2021 Update A Report from the American Heart Association. Lippincott Williams and Wilkins; 2021. 10.1161/CIR.0000000000000950 PMC13036842 33501848

[5] ONS. Mortality statistics. Official Labour Market Statistics 2020.

[6] Silverstein MD, Heit JA, Mohr DN, Petterson TM, O’Fallon WM, Melton LJ (1998). Trends in the Incidence of Deep Vein Thrombosis and Pulmonary Embolism: A 25-Year Population-Based Study. Arch Intern Med.

[7] What is Venous Thromboembolism? | CDC n.d. https://www.cdc.gov/ncbddd/dvt/facts.html (Accessed 23 Sept 2021).

[8] Giustozzi M, Franco L, Vedovati MC, Becattini C, Agnelli G (2019). Safety of direct oral anticoagulants versus traditional anticoagulants in venous thromboembolism. J Thromb Thrombolysis.

[9] Samuelson Bannow BT, Konkle BA (2018). Laboratory biomarkers for venous thromboembolism risk in patients with hematologic malignancies: A review. Thromb Res.

[10] Klovaite J, Benn M, Nordestgaard BG (2014). Obesity as a causal risk factor for deep venous thrombosis: a Mendelian randomization study. J Intern Med.

[11] Davey Smith G, Ebrahim S, Smith GD, Ebrahim S (2003). ‘Mendelian randomization’: can genetic epidemiology contribute to understanding environmental determinants of disease?. Int J Epidemiol.

[12] Evans DM, Davey SG (2015). Mendelian Randomization: New Applications in the Coming Age of Hypothesis-Free Causality. Annu Rev Genomics Hum Genet.

[13] Smith GD, Ebrahim S (2004). Mendelian randomization: prospects, potentials, and limitations. Int J Epidemiol.

[14] Hemani G, Zheng J, Elsworth B, Wade KH, Haberland V, Baird D, et al. The MR-base platform supports systematic causal inference across the human phenome. Elife 2018;7. 10.7554/eLife.34408 PMC5976434 29846171

[15] Zheng J, Baird D, Borges M-C, Bowden J, Hemani G, Haycock P (2017). Recent Developments in Mendelian Randomization Studies. Curr Epidemiol Rep.

[16] Yavorska OO, Burgess S (2017). MendelianRandomization: an R package for performing Mendelian randomization analyses using summarized data. Int J Epidemiol.

[17] Lawlor DA (2016). Commentary: Two-sample Mendelian randomization: opportunities and challenges. Int J Epidemiol.

[18] Goudswaard LJ, Bell JA, Hughes DA, Corbin LJ, Walter K, Davey Smith G (2021). Effects of adiposity on the human plasma proteome: observational and Mendelian randomisation estimates. Int J Obes.

[19] Zaghlool SB, Sharma S, Molnar M, Matías-García PR, Elhadad MA, Waldenberger M, et al. Revealing the role of the human blood plasma proteome in obesity using genetic drivers. Nature Communications 2021:1 2021;12:1–13. 10.1038/s41467-021-21542-4 PMC7904950 33627659

[20] Skrivankova VW, Richmond RC, Woolf BAR, Davies NM, Swanson SA, Vanderweele TJ, et al. Strengthening the reporting of observational studies in epidemiology using mendelian randomisation (STROBE-MR): explanation and elaboration. BMJ 2021;375. 10.1136/BMJ.N2233. 10.1136/bmj.n2233 PMC8546498 34702754

[21] Millard LAC, Davies NM, Gaunt TR, Davey Smith G, Tilling K (2018). Software Application Profile: PHESANT: a tool for performing automated phenome scans in UK Biobank. Int J Epidemiol.

[22] Bycroft C, Freeman C, Petkova D, Band G, Elliott LT, Sharp K (2018). The UK Biobank resource with deep phenotyping and genomic data. Nature.

[23] Haycock PC, Burgess S, Wade KH, Bowden J, Relton C, Davey SG (2016). Best (but oft-forgotten) practices: the design, analysis, and interpretation of Mendelian randomization studies. Am J Clin Nutr.

[24] Purcell S, Neale B, Todd-Brown K, Thomas L, Ferreira MAR, Bender D (2007). PLINK: A tool set for whole-genome association and population-based linkage analyses. Am J Hum Genet.

[25] Auton A, Abecasis GR, Altshuler DM, Durbin RM, Bentley DR, Chakravarti A (2015). A global reference for human genetic variation. Nature.

[26] Wu L, Candille SI, Choi Y, Xie D, Jiang L, Li-Pook-Than J (2013). Variation and Genetic Control of Protein Abundance in Humans. Nature.

[27] Battle A, Khan Z, Wang SH, Mitrano A, Ford MJ, Pritchard JK (2015). Impact of Regulatory Variation from RNA to Protein. Science.

[28] Sun BB, Maranville JC, Peters JE, Stacey D, Staley JR, Blackshaw J (2018). Genomic atlas of the human plasma proteome. Nature.

[29] Folkersen L, Fauman E, Sabater-Lleal M, Strawbridge RJ, Frånberg M, Sennblad B (2017). Mapping of 79 loci for 83 plasma protein biomarkers in cardiovascular disease. PLoS Genet.

[30] Suhre K, Arnold M, Bhagwat AM, Cotton RJ, Engelke R, Raffler J (2017). Connecting genetic risk to disease end points through the human blood plasma proteome. Nat Commun.

[31] Yao C, Chen G, Song C, Keefe J, Mendelson M, Huan T (2018). Genome-wide mapping of plasma protein QTLs identifies putatively causal genes and pathways for cardiovascular disease. Nat Commun.

[32] Wootton RE, Sallis HM (2020). Let’s call it the effect allele: a suggestion for GWAS naming conventions. Int J Epidemiol.

[33] Hemani G, Zheng J, Wade KH, Laurin C, Elsworth B, Burgess S, et al. MR-Base: a platform for systematic causal inference across the phenome using billions of genetic associations. BioRxiv 2016.

[34] Burgess S, Dudbridge F, Thompson SG (2016). Combining information on multiple instrumental variables in Mendelian randomization: comparison of allele score and summarized data methods. Stat Med.

[35] Lawlor Debbie A, Harbord Roger M, Sterne Jonathan AC, Timpson N, Davey Smith G, DA L (2008). Mendelian randomization: Using genes as instruments for making causal inferences in epidemiology. Stat Med..

[36] Yang J, Lee SH, Goddard ME, Visscher PM (2011). GCTA: a tool for genome-wide complex trait analysis. Am J Hum Genet.

[37] Yang J, Ferreira T, Morris AP, Medland SE, Genetic Investigation of AnTC, Consortium DiaIaGRAM (DIAGRAM) (2012). Conditional and joint multiple-SNP analysis of GWAS summary statistics identifies additional variants influencing complex traits. Nat Genet.

[38] Lyon MS, Andrews SJ, Elsworth B, Gaunt TR, Hemani G, Marcora E. The variant call format provides efficient and robust storage of GWAS summary statistics. Genome Biology 2021 22:1 2021;22:1–10. 10.1186/S13059-020-02248-0. 10.1186/s13059-020-02248-0 PMC7805039 33441155

[39] Boyd A, Golding J, Macleod J, Lawlor DA, Fraser A, Henderson J (2013). Cohort Profile: The ‘Children of the 90s’—the index offspring of the Avon Longitudinal Study of Parents and Children. Int J Epidemiol.

[40] Fraser A, Macdonald-Wallis C, Tilling K, Boyd A, Golding J, Davey Smith G (2013). Cohort Profile: The Avon Longitudinal Study of Parents and Children: ALSPAC mothers cohort. Int J Epidemiol.

[41] Yang Q, Sanderson E, Tilling K, Borges MC, Lawlor DA (2022). Exploring and mitigating potential bias when genetic instrumental variables are associated with multiple non-exposure traits in Mendelian randomization. Eur J Epidemiol.

[42] Giambartolomei C, Vukcevic D, Schadt EE, Franke L, Hingorani AD, Wallace C (2014). Bayesian Test for Colocalisation between Pairs of Genetic Association Studies Using Summary Statistics. PLoS Genet.

[43] Gregson J, Kaptoge S, Bolton T, Pennells L, Willeit P, Burgess S (2019). Cardiovascular Risk Factors Associated With Venous Thromboembolism. JAMA Cardiol.

[44] Zeng H, Lin C, Wang S, Zheng Y, Gao X (2021). Genetically predicted body composition in relation to cardiometabolic traits: a Mendelian randomization study. Eur J Epidemiol.

[45] Roetker NS, Armasu SM, Pankow JS, Lutsey PL, Tang W, Rosenberg MA (2017). Taller height as a risk factor for venous thromboembolism: a Mendelian randomization meta-analysis. J Thromb Haemost.

[46] Cushman M, O’Meara ES, Heckbert SR, Zakai NA, Rosamond W, Folsom AR (2016). Body size measures, hemostatic and inflammatory markers and risk of venous thrombosis: The Longitudinal Investigation of Thromboembolism Etiology. Thromb Res.

[47] Samama M-M, Group for the SS (2000). An Epidemiologic Study of Risk Factors for Deep Vein Thrombosis in Medical Outpatients: The Sirius Study. Arch Intern Med.

[48] Srisawat S, Sitasuwan T, Ungprasert P (2019). Increased risk of venous thromboembolism among patients with hyperthyroidism: a systematic review and meta-analysis of cohort studies. Eur J Intern Med.

[49] Horacek J, Maly J, Svilias I, Smolej L, Cepkova J, Vizda J (2015). Prothrombotic changes due to an increase in thyroid hormone levels. Eur J Endocrinol.

[50] Mousa SS, Davis FB, Davis PJ, Mousa SA. Human Platelet Aggregation and Degranulation Is Induced In Vitro by L-Thyroxine, but Not by 3,5,3′-Triiodo-L-Thyronine or Diiodothyropropionic Acid (DITPA): 2009;16:288–93. 10.1177/1076029609348315. 10.1177/1076029609348315 19903697

[51] Chang SLSW, Hu S, Huang YL, Lee MC, Chung WH, Cheng CY, et al. Treatment of Varicose Veins Affects the Incidences of Venous Thromboembolism and Peripheral Artery Disease. Circ Cardiovasc Interv 2021. 10.1161/CIRCINTERVENTIONS.120.010207 33685215

[52] Müller B, Leutgeb, Engeser, Achankeng N, Szecsenyi, Laux. Varicose veins are a risk factor for deep venous thrombosis in general practice patients. Vasa 2012;41:360–5. 10.1024/0301-1526/a000222. 10.1024/0301-1526/a000222 22915533

[53] Bertoletti L, Couturaud F (2021). COPD is not only one of the several VTE risk factors. Eur J Intern Med.

[54] Lankeit M, Held M. Incidence of venous thromboembolism in COPD: linking inflammation and thrombosis? n.d. 10.1183/13993003.01679-2015. 10.1183/13993003.01679-2015 26828045

[55] Kaze AD, Bigna JJ, Nansseu JR, Noubiap JJ (2018). Body size measures and risk of venous thromboembolism: protocol for a systematic review and meta-analysis. BMJ Open.

[56] Thaler E, Lechner K (1981). Antithrombin III Deficiency and Thromboembolism. Clin Haematol.

[57] Tang J, Zhu W, Mei X, Zhang Z (2018). Plasminogen activator inhibitor-1: A risk factor for deep vein thrombosis after total hip arthroplasty. J Orthop Surg Res.

[58] Maki RG (2010). Small Is Beautiful: Insulin-Like Growth Factors and Their Role in Growth, Development, and Cancer. J Clin Oncol.

[59] Fashanu OE, Heckbert SR, Aguilar D, Jensen PN, Ballantyne CM, Basu S (2017). Galectin-3 and venous thromboembolism incidence: the Atherosclerosis Risk in Communities (ARIC) Study. Res Pract Thromb Haemost.

[60] Klarin D, Busenkell E, Judy R, Lynch J, Levin M, Haessler J (2019). Genome-wide association analysis of venous thromboembolism identifies new risk loci and genetic overlap with arterial vascular disease. Nature Genetics.

[61] Klarin D, Emdin CA, Natarajan P, Conrad MF, Kathiresan S, Consortium I (2017). Genetic Analysis of Venous Thromboembolism in UK Biobank Identifies the ZFPM2 Locus and Implicates Obesity as a Causal Risk Factor. Circ Cardiovasc Genet.

[62] Isogai C, Laug WE, Shimada H, Declerck PJ, Stins MF, Durden DL (2001). Plasminogen activator inhibitor-1 promotes angiogenesis by stimulating endothelial cell migration toward fibronectin. Cancer Res.

[63] Hjortland GO, Lillehammer T, Somme S, Wang J, Halvorsen T, Juell S (2004). Plasminogen activator inhibitor-1 increases the expression of VEGF in human glioma cells. Exp Cell Res.

[64] Zhang Q, Lei L, Jing D (2020). Knockdown of SERPINE1 reverses resistance of triple-negative breast cancer to paclitaxel via suppression of VEGFA. Oncol Rep.

[65] Zhang Q, Zhang X, Zhang J, Wang B, Tian Q, Meng X (2022). Vascular endothelial growth factor and the risk of venous thromboembolism: a genetic correlation and two-sample Mendelian randomization study. Thromb J.

[66] Frischmuth T, Hindberg K, Aukrust P, Ueland T, Brækkan SK, Hansen JB (2022). Elevated plasma levels of plasminogen activator inhibitor-1 are associated with risk of future incident venous thromboembolism. J Thromb Haemost.

[67] Mo JW, Zhang DF, Ji GL, Liu XZ, Fan B (2015). TGF-β1 and serpine 1 expression changes in traumatic deep vein thrombosis. Genet Mol Res.

[68] Shimomura I, Funahashi T, Takahashi M, Maeda K, Kotani K, Nakamura T (1996). Enhanced expression of PAI–1 in visceral fat: Possible contributor to vascular disease in obeisty. Nature Medicine.

[69] Gadekar T, Dudeja P, Basu I, Vashisht S, Mukherji S (2020). Correlation of visceral body fat with waist–hip ratio, waist circumference and body mass index in healthy adults: A cross sectional study. Med J Armed Forces India.

[70] Sillen M, Declerck PJ (2020). Targeting PAI-1 in Cardiovascular Disease: Structural Insights Into PAI-1 Functionality and Inhibition. Front Cardiovasc Med.

[71] Laumen H, Skurk T, Hauner H (2008). The HMG-CoA reductase inhibitor rosuvastatin inhibits plasminogen activator inhibitor-1 expression and secretion in human adipocytes. Atherosclerosis.

[72] Glynn RJ, Danielson E, Fonseca FA, Genest J, Gotto AM, Kastelein JJ (2009). A Randomized Trial of Rosuvastatin in the Prevention of Venous Thromboembolism: the JUPITER Trial. N Engl J Med.

[73] Schol-Gelok S, de Maat MPM, Biedermann JS, van Gelder T, Leebeek FWG, Lijfering WM (2020). Rosuvastatin use increases plasma fibrinolytic potential: a randomised clinical trial. Br J Haematol.

[74] Jückstock J, Kimmich T, Mylonas I, Friese K, Dian D (2013). The inhibin-βC subunit is down-regulated, while inhibin-βE is up-regulated by interferon-β1a in Ishikawa carcinoma cell line. Arch Gynecol Obstet.

[75] Thomas TZ, Chapman SM, Hong W, Gurusingfhe C, Mellor SL, Fletcher R (1998). Inhibins, Activins, and Follistatins: Expression of mRNAs and Cellular Localization in Tissues From Men With Benign Prostatic Hyperplasia. Prostate.

[76] Détriché G, Gendron N, Philippe A, Gruest M, Billoir P, Rossi E (2022). Gonadotropins as novel active partners in vascular diseases: Insight from angiogenic properties and thrombotic potential of endothelial colony-forming cells. J Thromb Haemost.

[77] LaFoya B, Munroe JA, Mia MM, Detweiler MA, Crow JJ, Wood T (2016). Notch: A multi-functional integrating system of microenvironmental signals. Dev Biol.

[78] Mancarella S, Serino G, Dituri F, Cigliano A, Ribback S, Wang J (2020). Crenigacestat, a selective NOTCH1 inhibitor, reduces intrahepatic cholangiocarcinoma progression by blocking VEGFA/DLL4/MMP13 axis. Cell Death Differ.

[79] Casulo C, Ruan J, Dang NH, Gore L, Diefenbach C, Beaven AW (2016). Safety and Preliminary Efficacy Results of a Phase I First-in-Human Study of the Novel Notch-1 Targeting Antibody Brontictuzumab (OMP-52M51) Administered Intravenously to Patients with Hematologic Malignancies. Blood.

[80] Zhou Y, Zhang Y, Lian X, Li F, Wang C, Zhu F (2022). Therapeutic target database update 2022: facilitating drug discovery with enriched comparative data of targeted agents. Nucleic Acids Res.

[81] Davey Smith G, Hemani G (2014). Mendelian randomization: genetic anchors for causal inference in epidemiological studies. Hum Mol Genet.

[82] Hemani G, Tilling K, Davey Smith G. Orienting the causal relationship between imprecisely measured traits using GWAS summary data. PLoS Genet 2017;13. 10.1371/JOURNAL.PGEN.1007081. 10.1371/journal.pgen.1007081 PMC5711033 29149188

[83] Sakornsakolpat P, Prokopenko D, Lamontagne M, Reeve NF, Guyatt AL, Jackson VE (2019). Genetic landscape of chronic obstructive pulmonary disease identifies heterogeneous cell type and phenotype associations. Nat Genet.

[84] Konstantinides SV, Torbicki A, Agnelli G, Danchin N, Fitzmaurice D, Galiè N (2014). 2014 ESC Guidelines on the diagnosis and management of acute pulmonary embolismThe Task Force for the Diagnosis and Management of Acute Pulmonary Embolism of the European Society of Cardiology (ESC)Endorsed by the European Respiratory Society (ERS). Eur Heart J.

