# Supplementary material for: A phenome-wide approach to identify causal risk factors for deep vein thrombosis
Source: BMC Med Genomics. 2023 Nov 11;16:284. doi: 10.1186/s12920-023-01710-9 (PMC10640748; doi:10.1186/s12920-023-01710-9)
Supplement: Supplementary file 3 — Additional file 3. Supplementary Methods. [file 12920_2023_1710_MOESM3_ESM.docx]

# **Supplementary Methods**

## Protein quantitative trait locus (pQTL) data

**Goudswaard et al.** [1]**:** Used data for the MR analysis of BMI to protein levels from the prospective cohort study INTERVAL, which included data on both BMI and circulating proteome [2]. The sample-size of the study was 2422–2737 [1]. A polygenic risk score (PRS) for BMI was derived from SNPs associated with BMI generated by Yengo et al [3]. A one-sample MR using two-stage least squares (2SLS) regression was then done between the BMI PRS and protein measurements, adjusting for sex, age, smoking and alcohol drinking frequency [1]. The results from the MR were interpreted as a normalised SD-unit difference in each protein trait per normalised SD-unit (4.8 kg/m^2^) increase in BMI [1].

**Zaghlool et al.** [4]**:** Used data for the MR analysis of BMI to protein levels from the KORA F4 study done on participants from Southern Germany [4]. The sample-size of the study was 996. A PRS for BMI was derived from SNPs associated with BMI generated by Locke et al [5]. A one-sample MR using two-stage least squares (2SLS) regression was then done between the BMI PRS and protein measurements, adjusting for sex and age [4].

## Mendelian Randomization Analyses

**Two-sample MR sensitivity analyses:** Horizontal pleiotropy occurs when a SNP influences the outcome via a pathway other than the exposure of interest, thus violating a key assumption of MR (see **Supplementary Figure 1** [6]). MR methods which make differing assumptions regarding pleiotropy were performed as sensitivity analyses where genetic instruments were comprised of more than 3 SNPs: MR-Egger regression (each SNP is associated with the exposure independently of its pleiotropic effect), simple mode (across all SNPs effect estimates, the mode is 0), weighted mode (similar to simple mode, where weights are given to ratio estimates), and weighted median (at least 50% of all SNPs are valid instruments and no SNP contributes more than 50% of the weight; weight given to ratio estimates) [7–10].

While conventional MR methods assume effect homogeneity, large numbers of genetic instruments associated with an exposure can describe heterogenous effects (e.g. variants associated with BMI may be associated with DVT via a number of alterations to the circulating proteome) [11]. To test for genetic heterogeneity, we used the maximum likelihood [12] estimator and MR-Egger [8] for the exposures which were proxied by 2 or more variants. Heterogeneity of instrument effect estimates could indicate violations of MR assumptions due to horizontal pleiotropy, which can bias MR estimates [8,9]. At the same time, heterogeneity can indicate a more complex biological mechanism through which the exposure affects the outcome, indicating vertical pleiotropy, which does not invalidate MR assumptions [8,9].

**Two-sample MR mediation analysis:** The effect of BMI-associated proteins on DVT was estimated using the TwoSampleMR R package [13]. An IVW MR analysis was performed for FABP4, for which 3 SNPs were available to use as instruments. Wald ratios were derived for the remaining proteins. Where proteins were estimated to have a causal effect on DVT, a MR mediation analysis was performed to estimate the proportion mediated by a protein in the BMI-DVT link [14]. The method we used to calculate the proportion mediated by each protein was the product of coefficients method [14]. Here, the effect of BMI on protein levels is estimated, after which another MR is done to estimate the effect of protein levels on DVT. These are then multiplied to get the indirect effect, which is then divided by the total effect (BMI to DVT) to estimate the proportion mediated (**Figure 6‑4**) [14]. This assumes that the indirect and total effect are in the same direction (both negative or both positive) [14].

**Multiple testing correction:** As our MR-PheWAS estimated the causal relationship between a large number of exposures and DVT, we used PhenoSpD to estimate the number of independent traits in order to correct for multiple testing [15], which can adjust the P-value significance threshold better than through traditional methods, especially when many of traits are being tested. We used GWAS summary data describing the top 1000 associated SNPs for each exposure to create a phenotypic correlation matrix by Pearson correlation. This correlation matrix was used as an input for PhenoSpD to assess the number of independent exposures through matrix spectral decomposition [16,17], generating a P-value threshold of 5.43e-5. For the pQTL analysis, we used a Bonferroni correction, accounting for 15 independent tests (P=0.003), which would yield a similar threshold to a FDR correction given the small number of independent tests.

**Beta coefficient transformation:** Linear mixed model (LMM) methodology has gained popularity in GWAS due to its ability to control for population structure and deal with large datasets [18]. Regression (beta) coefficients from MR analyses are usually converted to odds ratios (ORs) or risk ratios (RRs) to make results interpretable. However, as GWAS software such as BOLT-LMM still use a linear model (rather than a logistic model) when analysing case-control traits, beta coefficients cannot be calculated directly when using thus must be approximated [18]. Using previously described methodology [19], we approximated logRRs for our LMM-derived MR estimates with the following formula: β / (μ * (1 - μ)), where μ = case fraction.

**Bidirectional MR:** Where there was evidence of an association with exposures tested in the MR-PheWAS, we performed a bidirectional MR analysis to assess the direction causality between a given exposure and DVT. This was conducted to identify potential pathways of reverse causation, which would invalidate MR assumptions [20].

[1] Goudswaard LJ, Bell JA, Hughes DA, Corbin LJ, Walter K, Davey Smith G, et al. Effects of adiposity on the human plasma proteome: observational and Mendelian randomisation estimates. International Journal of Obesity 2021 2021;6:1–9. https://doi.org/10.1038/s41366-021-00896-1.

[2] Di Angelantonio E, Thompson SG, Kaptoge S, Moore C, Walker M, Armitage J, et al. Efficiency and safety of varying the frequency of whole blood donation (INTERVAL): a randomised trial of 45 000 donors. Lancet 2017;390:2360. https://doi.org/10.1016/S0140-6736(17)31928-1.

[3] Yengo L, Sidorenko J, Kemper KE, Zheng Z, Wood AR, Weedon MN, et al. Meta-analysis of genome-wide association studies for height and body mass index in ∼700000 individuals of European ancestry. Hum Mol Genet 2018;27:3641. https://doi.org/10.1093/HMG/DDY271.

[4] Zaghlool SB, Sharma S, Molnar M, Matías-García PR, Elhadad MA, Waldenberger M, et al. Revealing the role of the human blood plasma proteome in obesity using genetic drivers. Nature Communications 2021 12:1 2021;12:1–13. https://doi.org/10.1038/s41467-021-21542-4.

[5] Locke AE, Kahali B, Berndt SI, Justice AE, Pers TH, Day FR, et al. Genetic studies of body mass index yield new insights for obesity biology. Nature 2015;518:197–206. https://doi.org/10.1038/nature14177.

[6] Davey Smith G, Hemani G. Mendelian randomization: genetic anchors for causal inference in epidemiological studies. Hum Mol Genet 2014;23:R89–98. https://doi.org/10.1093/hmg/ddu328.

[7] Hemani G, Bowden J, Haycock PC, Zheng J, Davis O, Flach P, et al. Automating Mendelian randomization through machine learning to construct a putative causal map of the human phenome. BioRxiv 2017.

[8] Bowden J, Davey Smith G, Burgess S. Mendelian randomization with invalid instruments: effect estimation and bias detection through Egger regression. Int J Epidemiol 2015;44:512–25. https://doi.org/10.1093/ije/dyv080.

[9] Hartwig FP, Davey Smith G, Bowden J. Robust inference in summary data Mendelian randomization via the zero modal pleiotropy assumption. Int J Epidemiol 2017;46:1985–98. https://doi.org/10.1093/ije/dyx102.

[10] Bowden J, Davey Smith G, Haycock PC, Burgess S. Consistent Estimation in Mendelian Randomization with Some Invalid Instruments Using a Weighted Median Estimator. Genet Epidemiol 2016;40:304–14. https://doi.org/https://doi.org/10.1002/gepi.21965.

[11] Bowden J, Spiller W, Del Greco M F, Sheehan N, Thompson J, Minelli C, et al. Improving the visualization, interpretation and analysis of two-sample summary data Mendelian randomization via the Radial plot and Radial regression. Int J Epidemiol 2018;47:1264–78. https://doi.org/10.1093/ije/dyy101.

[12] Pierce BL, Burgess S. Efficient Design for Mendelian Randomization Studies: Subsample and 2-Sample Instrumental Variable Estimators. Am J Epidemiol 2013;178:1177. https://doi.org/10.1093/AJE/KWT084.

[13] Hemani G, Zheng J, Wade KH, Laurin C, Elsworth B, Burgess S, et al. MR-Base: a platform for systematic causal inference across the phenome using billions of genetic associations. BioRxiv 2016.

[14] Carter AR, Sanderson E, Hammerton G, Richmond RC, Davey Smith G, Heron J, et al. Mendelian randomisation for mediation analysis: current methods and challenges for implementation. European Journal of Epidemiology 2021 36:5 2021;36:465–78. https://doi.org/10.1007/S10654-021-00757-1.

[15] Zheng J, Richardson T, Millard L, Hemani G, Raistrick C, Vilhjalmsson B, et al. PhenoSpD: an atlas of phenotypic correlations and a multiple testing correction for the human phenome. BioRxiv 2017. https://doi.org/10.1101/148627.

[16] Nyholt DR. A simple correction for multiple testing for single-nucleotide polymorphisms in linkage disequilibrium with each other. Am J Hum Genet 2004;74:765–9. https://doi.org/10.1086/383251.

[17] Li J, Ji L. Adjusting multiple testing in multilocus analyses using the eigenvalues of a correlation matrix. Heredity (Edinb) 2005;95:221–7. https://doi.org/10.1038/sj.hdy.6800717.

[18] Loh PR, Tucker G, Bulik-Sullivan BK, Vilhjálmsson BJ, Finucane HK, Salem RM, et al. Efficient Bayesian mixed-model analysis increases association power in large cohorts. Nat Genet 2015;47:284–90. https://doi.org/10.1038/ng.3190.

[19] Lloyd-Jones LR, Robinson MR, Yang J, Visscher PM. Transformation of Summary Statistics from Linear Mixed Model Association on All-or-None Traits to Odds Ratio. Genetics 2018;208:1397.

[20] Zheng J, Baird D, Borges M-C, Bowden J, Hemani G, Haycock P, et al. Recent Developments in Mendelian Randomization Studies. Curr Epidemiol Rep 2017;4:330–45. https://doi.org/10.1007/s40471-017-0128-6.
